# Duration of Antimicrobial Treatment in Adult Patients with Pneumonia: A Narrative Review

**DOI:** 10.3390/antibiotics13111078

**Published:** 2024-11-12

**Authors:** Dimitra Dimopoulou, Charalampos D. Moschopoulos, Konstantina Dimopoulou, Anastasia Dimopoulou, Maria M. Berikopoulou, Ilias Andrianakis, Sotirios Tsiodras, Anastasia Kotanidou, Paraskevi C. Fragkou

**Affiliations:** 1 Second Department of Pediatrics, “Aghia Sophia” Children’s Hospital, 11527 Athens, Greece; dimi_med@hotmail.com (D.D.); maria-beri@med.uoa.gr (M.M.B.); 2 Fourth Department of Internal Medicine, School of Medicine, Attikon University Hospital, National and Kapodistrian University of Athens, 12462 Athens, Greece; bmosxop@yahoo.gr (C.D.M.); sotirios.tsiodras@gmail.com (S.T.); 3 Department of Gastroenterology, Red Cross Hospital of Athens, 11526 Athens, Greece; conu_med@hotmail.com; 4 Department of Pediatric Surgery, “Aghia Sophia” Children’s Hospital, 11527 Athens, Greece; natasa_dimo@hotmail.com; 5 Department of Intensive Care Unit, Hygeia Hospital, 15123 Athens, Greece; iandri@med.uoa.gr; 6 First Department of Critical Care Medicine and Pulmonary Services, School of Medicine, Evangelismos Hospital, National and Kapodistrian University of Athens, 10676 Athens, Greece; akotanid@med.uoa.gr

**Keywords:** duration, antibiotics, community-acquired pneumonia, hospital-acquired pneumonia, ventilator-associated pneumonia

## Abstract

Pneumonia remains a major global health concern, causing significant morbidity and mortality among adults. This narrative review assesses the optimal duration of antimicrobial treatment in adults with community-acquired pneumonia (CAP), hospital-acquired pneumonia (HAP), and ventilator-associated pneumonia (VAP). Current evidence about the impact of treatment duration on clinical outcomes demonstrates that shorter antibiotic courses are non-inferior, regarding safety and efficacy, compared to longer courses, particularly in patients with mild to moderate CAP, which is in line with the recommendations of international guidelines. Data are limited regarding the optimal antimicrobial duration in HAP patients, and it should be individually tailored to each patient, taking into account the causative pathogen and the clinical response. Shorter courses are found to be as effective as longer courses in the management of VAP, except for pneumonia caused by non-fermenting Gram-negative bacteria; however, duration should be balanced between the possibility of higher recurrence rates and the documented benefits with shorter courses. Additionally, the validation of reliable biomarkers or clinical predictors that identify patients who would benefit from shorter therapy is crucial. Insights from this review may lead to future research on personalized antimicrobial therapies in pneumonia, in order to improve patient outcomes.

## 1. Introduction

Pneumonia is defined as an acute infection of the lung parenchyma by various pathogens; its diagnosis is based on specific clinical, imaging, and laboratory criteria and represents a leading cause of morbidity and mortality globally [1,2,3]. Based on the mode that is acquired, it is classified as community-acquired pneumonia (CAP) and hospital-acquired pneumonia (HAP) [1].

CAP represents the second most commonly reported cause of hospitalization. Evidence shows that CAP increases morbidity and mortality and healthcare costs [4]. HAP is the most common healthcare-associated infection in adults, representing up to 22% of all healthcare-associated infections, and it is further classified as non-ventilator hospital-acquired pneumonia (nvHAP) and ventilator-associated pneumonia (VAP) [5]. According to internationally published data, HAP is a leading cause of mortality among healthcare-associated infections, and it is associated with prolonged hospitalization and an increase in healthcare costs [6,7].

The treatment cornerstone of pneumonia is the administration of antimicrobial agents. Appropriate antimicrobial regimen, including the dose, the agent choice, and the duration, is essential to attain optimal patient outcomes. On the other hand, inappropriately prolonged duration and overly broad-spectrum antimicrobial therapy can increase the risk of *Clostridium difficile* infection and antibiotic-related adverse effects due to sustained exposure to antimicrobials and contribute, inevitably, to the development of multidrug-resistant organisms [8,9].

Although current evidence supports a shorter duration of antimicrobial therapy for bacterial pneumonia, especially for patients with uncomplicated CAP, prolonged administration of antimicrobials remains a common practice, raising the associated risks of excess antimicrobial treatment [10,11,12]. Reducing the duration of antimicrobial treatment in patients with pneumonia is crucial and could be established with appropriate antimicrobial stewardship interventions [13,14].

Although international guidelines for the management of CAP, VAP, and nvHAP have been published [15,16,17], there are significant gaps and controversies regarding the optimal duration of their antimicrobial treatment. The reduction in the treatment duration of these three different types of pneumonia is of great importance, because of the high rate of antimicrobial consumption and prevalence of multidrug-resistant organisms [18,19,20]. In this context, the present review gives an updated overview of the existing evidence regarding the developments and current status of the duration of antibiotic therapy in all different types of pneumonia, focusing on the efficacy and clinical outcomes of shorter courses of antibiotics compared with longer courses, in order to shed light on this controversial topic and identify opportunities of further improvement. In addition, this review aims to provide a comprehensive understanding of the potential benefits and limitations of adopting shorter antibiotic courses in clinical practice and offer insights into optimizing pneumonia treatment protocols to enhance patient outcomes and address the growing challenge of antibiotic resistance.

## 2. Current Guidelines on the Duration of Antimicrobial Treatment in Different Types of Pneumonia

There are several international guidelines referring to diagnostic, management, and therapeutic decisions of pneumonia in adults. Adherence to current guidelines may lead to shorter hospitalization, decreased antimicrobial resistance, and lower healthcare costs [15,16,17]. Due to the overuse of antibiotics with the inappropriate prescription and prolonged duration of treatment, there is a need to update guidelines with a shift towards a shorter approach for antibiotic duration. Several randomized controlled trials (RCTs) have been performed, but further research is still needed to define the optimal duration of antibiotic therapy. Current international guidelines for different types of pneumonia are summarized below. The definitions of the different types of pneumonia are presented in Table 1.

### 2.1. Community-Acquired Pneumonia

The management of CAP in adults varies among different guidelines, which are all summarized in Table 2.

British Thoracic Society (BTS) guidelines, published in 2001 and updated in 2004 and 2009, on the management of pneumonia in adults in the community or in hospital without predisposing conditions such as immunosuppression or cancer, report that the treatment and the optimal duration of antibiotic therapy depends on the severity assessment of pneumonia [26]. These guidelines state that, for most patients with low- or moderate-severity and uncomplicated pneumonia, 7 days of antibiotic treatment is recommended, whereas 7–10 days of therapy is suggested for patients with high-severity pneumonia. In the case of confirmed or suspected pathogens, such as Staphylococcus aureus or Gram-negative enteric bacilli, the optimal duration of antibiotic may be extended to 14 or 21 days [26]. However, more recent guidelines from the National Institute for Health and Care Excellence (NICE) published in 2019 and partially updated in 2023 suggest a 5-day treatment for all types of pneumonia, regardless of the severity, if clinical stability is achieved [27].

In 2019, the American Thoracic Society (ATS) and Infectious Diseases Society of America (IDSA) updated their previous guidelines [36] and provided evidence-based recommendations for the treatment of CAP in adults who have not recently traveled abroad and do not have an immunocompromising condition. Based on RCTs [36,37,38,39], these guidelines strongly recommend a minimum of 5 days of treatment for low-, moderate-, and high-severity CAP without infectious complications, even if clinical stability has been achieved earlier. In cases of proven or suspected Methicillin-resistant Staphylococcus aureus (MRSA) or *Pseudomonas aeruginosa*, the antibiotic therapy should be prolonged at 7 days [10].

The first international guidelines on the management of patients with severe CAP who needed Intensive Care Unit (ICU) admission, by the European Respiratory Society (ERS), European Society of Intensive Care Medicine (ESICM), European Society of Clinical Microbiology and Infectious Diseases (ESCMID), and Latin American Thoracic Association (Asociación Latinoamericana del Tórax) (ALAT), were published in 2023. Based on three RCTs using serum procalcitonin (PCT) as a biomarker for shorter antibiotic therapy [40,41,42], they concluded that clinical stability is achieved after 5–7 days of antibiotic therapy. Finally, these guidelines advocate that in patients with CAP caused by S. aureus, antibiotic therapy should be prolonged to a minimum of 7 days and cannot be shortened by PCT, which is in line with the IDSA guidelines [28].

### 2.2. Hospital-Acquired Pneumonia

The duration of antibiotic therapy in adults with HAP is generally longer than in those with CAP, reflecting the more resistant nature of hospital pathogens. Table 2 illustrates the management of HAP in adults according to various guidelines.

NICE guidelines, published in 2019, recommend specific antibiotic therapies based on the severity of symptoms and the risk of resistance. Risk factors for increased antibiotic resistance include symptoms starting after ≥5 days in hospital, severe lung disease or immunosuppression, recent use of broad-spectrum antibiotics, colonization with multidrug-resistant bacteria, and recent exposure to a healthcare environment before admission [27]. According to these guidelines, in HAP patients with mild symptoms and a low risk of resistance, the initial recommended antibiotic duration is 5 days. After the 5-day treatment period, they suggest reviewing the patient and discontinuing the treatment if clinical stability is achieved [27]. Furthermore, in patients with severe symptoms and signs of sepsis, as well as in HAP with a higher risk of resistance or suspected MRSA, the duration of the treatment is unclear. In any case, antibiotics should be reviewed after 5 days [27].

ATS/IDSA guidelines, published in 2016, 11 years after the previous ones, strongly recommend a 7-day antibiotic course. Due to inadequate studies comparing the longer versus shorter antibiotic duration in HAP patients, this recommendation was based on patients with VAP. It seems that a shorter duration reduces the risk of side effects such as *C. difficile* colitis [22]. ERS/ESICM/ESCMID/ALAT 2017 international guidelines also suggest a 7–8-day course regimen [29] based on RCTs conducted in VAP patients [43,44].

### 2.3. Ventilator-Associated Pneumonia

Guidelines for the management of VAP in adults are similar to those of HAP (Table 2).

The optimal duration of antibiotics treatment according to ATS/IDSA 2016 guidelines, based on RCTs and observational studies, is 7 days [22,43,45]. According to the 2017 guidelines by ERS/ESICM/ESCMID/ALAT, the duration of the antibiotic treatment in adults with VAP is suggested to be 7–8 days [29]. These guidelines apply to immunocompetent patients, without cystic fibrosis, lung abscess, empyema or necrotizing pneumonia. However, this suggestion of a 7–8-day course also refers to patients with non-fermenting Gram-negative bacteria (NF-GNB), *Acinetobacter* spp., and MRSA who exhibit a good clinical response [29].

## 3. Evidence on the Shorter vs. Longer Duration of Antibiotic Treatment in Different Types of Pneumonia

**Figure 1:**
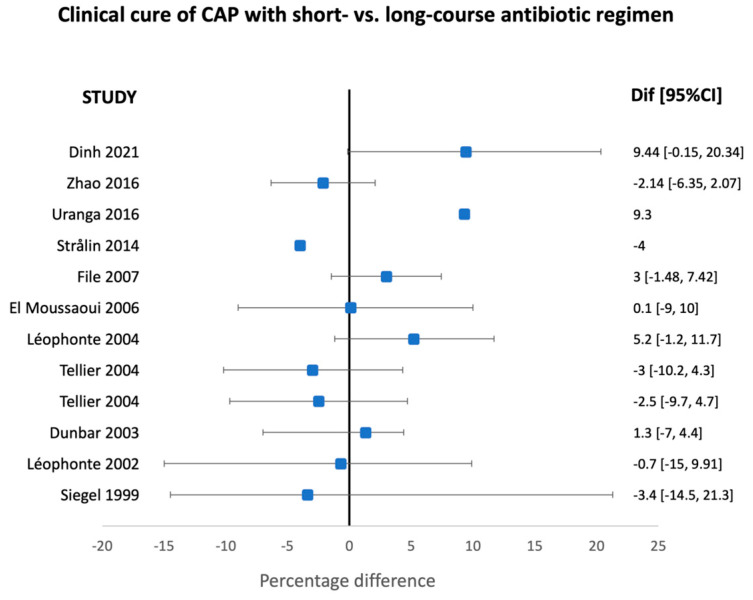
Clinical cure of adult patients with community-acquired pneumonia receiving short- versus long-course antibiotic regimens in the available randomized clinical trials [46,47,48,49,50,51,52,53,54,55,56].

**Figure 2:**
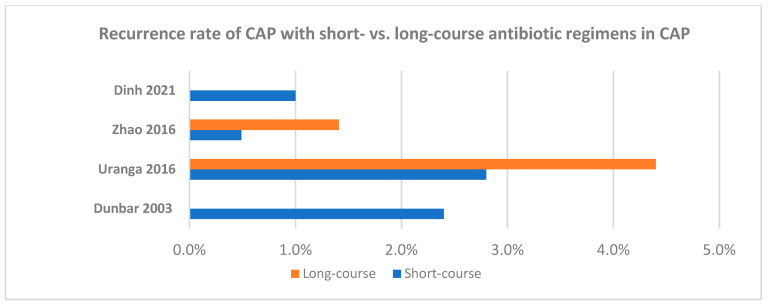
Recurrence rate of adult patients with community-acquired pneumonia receiving short- versus long-course antibiotic regimens in the available randomized clinical trials [46,47,48,54].

**Figure 3:**
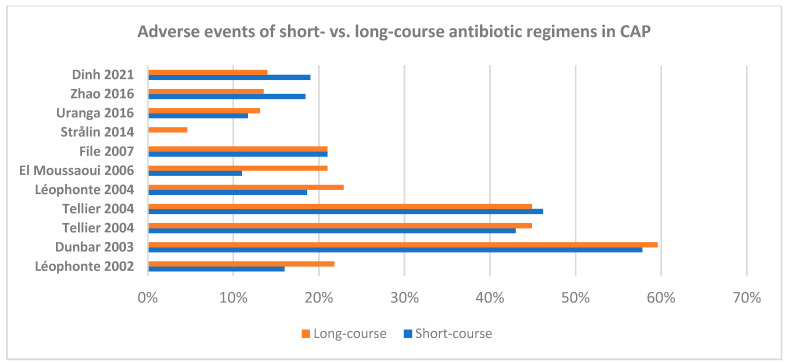
Adverse events of short- versus long-course antibiotic regimens in adult patients with community-acquired pneumonia, as retrieved from the available randomized clinical trials [46,48,50,52,53,55].

**Figure 4:**
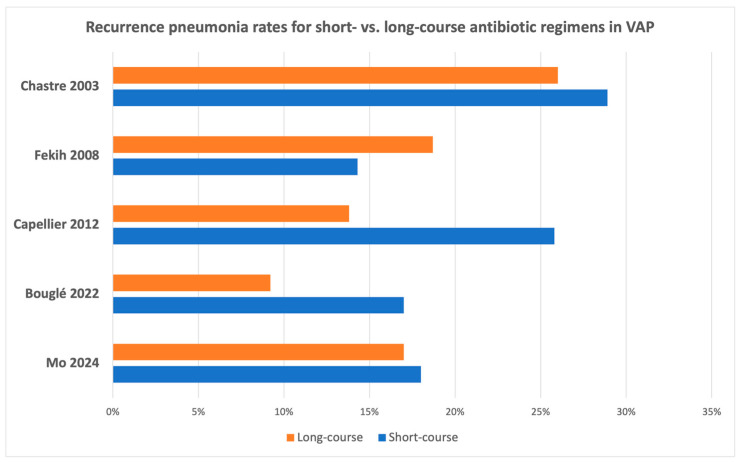
Recurrence rate of adult patients with ventilator-associated pneumonia receiving short- versus long-course antibiotic regimens in the available randomized clinical trials [44,57,58,59,60].

The characteristics of randomized clinical trials for short- versus long-course antibiotic regimens for the different types of pneumonia in adults are summarized in Table 3 and Table 4, as well as in Figure 1, Figure 2, Figure 3 and Figure 4.

**Table 3:** Summary of randomized clinical trials for short- vs. long-course antibiotic regimens for community-acquired pneumonia.

Author (Publication Year)	Participants—Treatment Groups	Primary Outcome	Results (Short vs. Long Course)	Comments
Siegel et al. (1999) [56]	46 inpatients with moderately severe CAP Short course = 24 (7 d: 2 d of IV cefuroxime 750 mg × 3 followed by 5 d of oral cefuroxime axetil 500 mg × 2) Long course = 22 (10 d: 2 days of IV cefuroxime 750 mg × 3 followed by 8 days of oral cefuroxime axetil 500 mg × 2)	Clinical cure at d42	87.5% vs. 90.9% (95% CI, −14.5% to 21.3%)	1. Confirmed non-inferiority for the short course 2. No late recurrence 3. No difference in the length of hospital stay 4. Potential US cost-savings: USD 27.2 million 5. Antibiotic side effects: mild and infrequent 6. The most commonly isolated microorganism was *Streptococcus pneumoniae* and none of them was penicillin resistant
Léophonte et al. (2002) [55]	186 inpatients with CAP Short course = 94 (5 d of IV Ceftriaxone 1 g × 1) Long course = 92 (10 d of IV Ceftriaxone 1 g × 1)	Clinical cure at EOT (d10)	81.9% vs. 82.6% (95% CI, −∞ to 9, 91%)	1. Confirmed non-inferiority for the short course 2. No difference in clinical normalization on day 10: 90.1% vs. 93.2% (95% CI, −∞ to 9, 81%) 3. No difference in clinical cure rate at follow-up (day 30–45): 73.4% vs. 72.8% [95% CI, −∞ to –0.58%] 4. No difference in radiological response rates at follow-up (day 30–45) 5. No difference in antibiotic side effects (16% vs. 21.8%) 6. No difference in liver toxicity. No nephrotoxicity 7. The most commonly isolated microorganism was followed by *Staphylococcus* spp.
Dunbar et al. (2003) [54]	390 inpatients and outpatients with mild to moderate CAP Short course = 198 (5 d of IV/oral levofloxacin 750 mg/d) Long course = 192 (10 d of IV/oral levofloxacine 500 mg/d)	Clinical cure at d7–14 post treatment	92.4% vs. 91.1% (95% CI, −7.0 to 4.4)	1. Confirmed non-inferiority for the short course 2. No difference in the bacteriological success rate and all-cause mortality 3. No difference in antibiotic side effects (57.8% vs. 59.6%) 4. The most commonly isolated bacteria were *Streptococcus pneumoniae, Mycoplasma pneumoniae and Haemophilus* spp. All of them are susceptible to levofloxacin 5. Clinical cure at d 7–14: *Streptococcus pneumoniae:* 90.9% vs. 90% *Haemophilus influenzae*: 92.3% vs. 92.9% *Haemophilus parainfluenzae*: 100% vs. 90% *Mycoplasma pneumoniae*: 95.3% vs. 94.4%
Tellier et al. (2004) [53]	575 inpatients and outpatients with mild to moderate CAP Short course = 193 (5 d of oral Telithromycin 800 mg × 1) or 195 (7 d of oral Telithromycin 800 mg × 1) Long course = 187 (10 d of oral Clarithromycin 500 mg × 2)	Clinical cure at EOT	89.3% (5 days) vs. 91.8% (10 days) (dif = 2.5 (−9.7, 4.7)	1. Confirmed non-inferiority for the short course 2. No difference in bacteriologic outcome rates 3. No difference in clinical efficacy against pneumococcal bacteremia and common respiratory pathogens, including macrolide-resistant isolates 4. Antibiotic side effects: mild—no difference [43% (5 days) vs. 46.2% (7 days) vs. 44.9% (10 days)] 5. No difference in liver toxicity. No nephrotoxicity 6. No difference in mortality 7. The most common bacteria were *Streptococcus pneumoniae*, *Haemophilus influenzae* and *Moraxella catarrhalis* 8. *Streptococcus pneumoniae* isolates: 8.7% resistant to erythromycin and 0% resistant to penicillin. Clinical cure in cases with macrolide-resistant isolates receiving short course of antibiotics: 100%
			88.8% (7 days) vs. 91.8% (10 days) (dif = 3.0 (−10.2, 4.3)	
Léophonte et al. (2004) [52]	249 inpatients with CAP of suspected *Streptococcus pneumoniae* origin Short course = 128 (7 d of oral gemifloxacin 320 mg × 1) Long course = 121 (10 d of oral amoxicillin/clavulanate 1 g/125 mg × 3)	Clinical cure at EOT (d12–14) and follow-up (d24–30)	EOT: 95.3% vs. 90.1 (95% CI, −1.2% to 11.7%) Follow-up: 88.7% vs. 87.6 (95% CI, −7.3% to 9.5%)	1. Confirmed non-inferiority for the short course 2. No difference in clinical cure in patients with severe CAP 3. No difference in bacteriologic response rates at EOT and at follow-up 4. No difference in radiological response rates at EOT and at follow-up 5. No difference in antibiotic side effects (18.6% vs. 22.9%) 6. Liver toxicity: 7.8% vs. 1.3%, but it was transient, with lower values at EOT. No nephrotoxicity 7. The most common microorganism was *Streptococcus pneumoniae,* followed by *Mycoplasma pneumoniae, Legionella pneumophila* and *Chlamydia pneumoniae* 8. Clinical cure at EOT: *Streptococcus pneumoniae:* 96% vs. 100% *Haemophilus influenzae*: 78% vs. 100% *Mycoplasma pneumoniae*: 94% vs. 83% *Legionella pneumophila*: 80% vs. 83% *Chlamydia pneumoniae*: 100% vs. 100% 9. No isolate was resistant to ofloxacin, and 5.8% of *Streptococcus pneumoniae* isolates were resistant to penicillin. All of them were eradicated by the antibiotic courses.
El Moussaoui et al. (2006) [51]	119 inpatients with mild to moderate/severe CAP (pneumonia severity index score < or = 110), who substantially improved after three days’ treatment Short course = 56 (3 d of IV Amoxicillin 1 g × 4) Long course = 63 (8 d: IV Amoxicillin 1 g × 4 for 3 d followed by oral amoxicillin 750 mg × 3 for 5 d)	Clinical cure at d10	93% vs. 93% (dif = 0.1%, 95% CI, −9% to 10%)	1. Confirmed non-inferiority for the short course 2. No difference in clinical cure on day 28: 90% vs. 88% (difference 2%, − 9% to 15%) 3. No difference in bacteriological and radiological success rates on days 10 and 28 4. No difference in clinical efficacy against pneumococcal bacteremia 5. Antibiotic side effects: mild, no difference (11% vs. 21%)
File et al. (2007) [50]	510 outpatients with mild to moderate CAP Short course = 256 (5 d of oral Gemifloxacin 320 mg × 1) Long course = 254 (7 d of oral Gemifloxacin 320 mg × 1)	Clinical cure at d24–30	95% vs. 92% (95% CI, −1.48 to 7.42)	1. Confirmed non-inferiority for the short course 2. No difference in clinical cure at EOT: 95.5% vs. 95.8% [95% CI, −3.85 to 3.42] 3. No difference in bacteriological and radiological success rates at EOT and follow-up 4. No difference in antibiotic side effects (21% vs. 21%) 5. Liver toxicity: elevated ALT 7.4% vs. 4.7% and elevated AST 7.4% vs. 2.8%. No nephrotoxicity. 6. The most common microorganism was *Streptococcus pneumoniae,* followed by *Chlamydia pneumoniae, Mycoplasma pneumoniae, Haemophilus influenzae* and *Staphylococcus aureus* 7. Multidrug-resistant *Streptococcus pneumoniae* was isolated in 28% (23% vs. 33%). 8. Eradication rate for *Streptococcus pneumoniae* was 100% vs. 95%, and for multidrug-resistant *Streptococcus pneumoniae*, it was 100% vs. 66.7%.
Strålin et al. (2014) [49]	174 inpatients with CAP Short course = 88 (at least 5 d of beta-lactam) Long course = 86 (10 d of beta-lactam)	Clinical cure at EOT	90% vs. 94%	1. Confirmed non-inferiority for the short course 2. No difference in clinical cure at pneumococcal CAP: 96% vs. 97% 3. No difference in antibiotic side effects (0% vs. 4.6%) 4. The most common microorganism was *Streptococcus pneumoniae*, and the clinical cure in these cases was 96% vs. 97%.
Uranga et al. (2016) [48]	312 inpatients with CAP Short course = 162 (median 5 d (IQR 5–6.5) of various antibiotics) Long course = 150 (median 10 d (IQR 10–11) of various antibiotics)	Clinical cure at d10 and d30	D10: 59.7% vs. 50.4% (*p* = 0.12) D30: 94.4% vs. 92.7% (*p* = 0.54)	1. Shorter courses based on clinical stability criteria can be safely implemented in hospitalized patients with CAP. 2. No difference between the different severity groups and types of antibiotics 3. No difference in in-hospital and 30-day mortality, in-hospital complications, recurrence by day 30, length of hospital stay, and radiologic resolution 4. Readmission by day 30 was significantly more common in the long course than in the short course (6.6% vs. 1.4%; *p* = 0.02). 5. No difference in antibiotic side effects (13.1% vs. 11.7%)
Zhao et al. (2016) [47]	427 inpatients and outpatients with mild to moderate CAP Short course = 208 (5 d of IV levofloxacin 750 mg/day) Long course = 219 (at least 7 d (range 7–14) of IV/oral levofloxacin 500 mg/d)	Clinical cure at EOT	93.8% vs. 95.9% (OR 0.643 (95% CI, 0.269, 1.537) (dif −2.14 (95% CI, −6.35, 2.07) *p* = 0.35	1. Confirmed non-inferiority for the short course 2. No difference in the bacteriological success rate 3. No difference in antibiotic side effects (18.42% vs. 13.54%). No difference in liver toxicity. No nephrotoxicity 4. The most commonly isolated microorganism was *Streptococcus pneumoniae*, and all the isolates were eradicated in both groups.
Dinh et al. (2021) [46]	310 inpatients with moderately severe CAP Short course = 157 (3 d of beta-lactam) Long course = 153 (8 d: beta-lactam for 3 days followed by oral amoxicillin 1 g plus clavulanate 125 mg × 3)	Clinical cure at d15	78% vs. 68% (dif = 9.44% [95% CI, −0.15 to 20.34])	1. Confirmed non-inferiority for the short course 2. No difference in clinical cure at day 30: 74% vs. 76% (dif –1.42%) (95% CI, –12.08 to 9.20) 3. No difference in mortality on day 30 and length of hospital stay 4. No difference between the different age and severity groups 5. No difference in antibiotic side effects (19% vs. 14%). No difference in liver toxicity. No nephrotoxicity

ALT: Alanine transaminase; AST: aspartate aminotransferase; CI: confidence interval; CAP: community-acquired pneumonia; dif: difference; EOT: end of therapy; IV: intravenous; OR: odds ratio; vs.: versus.

**Table 4:** Summary of randomized clinical trials for short- vs. long-course antibiotic regimens for hospital-acquired and ventilator-associated pneumonia.

Author (Publication Year)	Participants	Primary Outcome	Results (Short vs. Long Course)	Comments
Hospital-acquired pneumonia				
Singh et al. (2000) [61]	Short course = 39 (3 d) Usual care = 42 (9.8 d; 4–20)	3 d mortality 14 d mortality 30 d mortality	0% vs. 7 %, *p* > 0.05 8% vs. 21%, *p* > 0.05 13% vs. 31%, *p* > 0.05	1. Mixed case population with HAP (42%) and VAP (58%) patients 2. Confirmed non-inferiority for the short course 3. Shorter LOS in ICU for the short course 4. Lower cost of antimicrobial therapy for the short course 5. Lower antimicrobial resistance/superinfection rate for the short course
Ventilator-associated pneumonia				
Mo et al. (2024) [60] REGARD-VAP	Short course = 232 (6 d; 5–7) Usual care = 229 (14 d; 10–21)	Death or pneumonia recurrence (60 d)	41% vs. 44% (dif = −3%; −∞, 5%)	1. Confirmed non-inferiority, but not superiority 2. Antibiotic side effects = −31% (−37 to −25, *p* < 0.0001); kidney injury = −30% (−36 to −24, *p* < 0.0001); liver injury = −3% (−5 to −1, *p* = 0.033) 3. Composite outcome for NF-GNB VAP, OR = 1.38 (0.65 to 2.92, *p* = 0.40) 4. New CRE acquisition or infection, dif = 0.0009% (−0.061 to 0.059, *p* = 0.98)
Bouglé et al. (2022) [59] iDIAPASON	Short course = 88 (8 d) Long course = 98 (15 d) (VAP from *Ps. aeruginosa*)	Composite: mortality and VAP recurrence (90 d)	35.2% vs. 25.5 (dif = 9.7%; −1.9% −21.2%)	1. Did not confirm non-inferiority for the short course due to inadequate enrolment (target = 600) 2. New MDRO acquisition, dif = −4.5% (−16.8 to 8.3)
Kollef et al. (2012) [62]	Doripenem (7 d) = 115 Imipenem (10 d) = 112	Clinical cure at EOT (d10)	45.6% vs. 56.8%; 95% CI, −26.3% to 3.8%	1. 28 d mortality = 21.5% vs. 14.8%; 95% CI, −5.0 to 18.5 2. VAP relapse, hospital mortality, LOS were similar between groups
Capellier et al. (2012) [58]	Short course = 116 (8 d) Usual care = 109 (15 d) (early-onset VAP)	Clinical cure at d21	85.3% vs. 84.4% (dif = 0.9%; −8.4% −10.3%) OR = 0.929 (0.448–1.928)	1. Only early-onset VAP 2. Equivalence between the two arms 3. No dif in mortality 4. Secondary infection higher in 8 d cohort
Fekih et al. (2008) [57]	Short course = 14 (7 d) Long course = 16 (10 d)	14 d mortality 28 d mortality	7.1 vs. 35.7% (7 d) 31.2 vs. 37.5% (10 d)	1. No dif in mortality 2. No dif in recurrent pulmonary infection 3. No dif in ICU LOS
Chastre et al. (2003) [44] PneumoA	Short course = 197 (8 d) Long course = 204 (15 d)	28 d mortality 28 d recurrence Antibiotic-free days	18.8% vs. 17.2% (dif = 1.6%; −3.7%–6.9%) 28.9% vs. 26% (dif = 2.9%; −3.2%−9.1%) 13.1 vs. 8.7 days (*p* < 0.001)	1. No dif in 28 d mortality and recurrence rate 2. NF-GNB VAP: higher recurrence rate with short course 3. Less recurrences from MDRO in the short course

CI: confidence interval; CRE: carbapenem-resistant Enterobacterales; dif: difference; EOT: end of therapy; HAP: hospital-acquired pneumonia; ICU: Intensive Care Unit; LOS: length of stay; MDROs: multidrug-resistant organisms; NF-GNB: non-fermenting Gram-negative bacteria; OR: odds ratio; VAP: ventilator-associated pneumonia; vs.: versus.

### 3.1. Community-Acquired Pneumonia

The management of CAP in adults revolves around the appropriate duration of antibiotics, in order to decrease antimicrobial resistance of CAP-related pathogens, such as Streptococcus pneumoniae and Staphylococcus aureus [2]. The optimal duration of antimicrobial treatment remains unclear and controversial. However, several randomized controlled trials (RCTs) and observational studies have increasingly focused on comparing the efficacy (as defined by the clinical cure rates, microbiological eradication, and recurrence rates), the safety, and the adverse effects of short- versus long-course antibiotic regimens in adult patients with CAP.

#### 3.1.1. Evidence on Antibiotic Treatment Duration of CAP

The first meta-analysis was published in 2007 and included 15 RCTs comparing the outcome of short courses (7 days or less) versus extended courses (>7 days) of antibiotic monotherapy for CAP in adults [38]. This meta-analysis showed that there was no difference in the risk of clinical failure, bacteriologic eradication, or mortality between the short- and extended-course regimens [38]. The second meta-analysis was published in 2008 and evaluated only five RCTs involving outpatients and hospitalized adults with mild to moderate CAP, who did not require intensive care; in contrast to the first meta-analysis, this meta-analysis aimed to compare treatment with the same antibacterial agent, in the same daily dosages, but with different total duration of administration and excluded studies reporting on azithromycin, which is an antibiotic with a prolonged half-life [37]. The results demonstrated that there were no differences between short- (3–7 days) and long-course (7–10 days) regimens regarding clinical success at end-of-therapy and follow-up, microbiological success, relapses, mortality, and adverse events [37]. In addition, no differences were found in the clinical success of patients treated with no more than 5-day short-course regimens versus at least 7-day long-course regimens [37]. A third meta-analysis of non-duplicate data from 17 RCTs included in the previous two meta-analyses showed a non-significant difference in the rate of treatment failure between short- (≤7 days) and long- (>7 days) course antibiotic treatment, while the trial sequential analysis suggested that further trials on this topic would not affect current evidence [63].

Another meta-analysis, including 21 clinical trials (overlapping with the previous meta-analyses) and 4861 patients (19 out of 21 trials were RCTs), indicated that the clinical cure was similar between the short-course (≤6 days) and long-course (≥7 days) treatment in adults with CAP, irrespective of patient setting (outpatient or inpatient) and severity of pneumonia [39]. Also, relapses and antibiotic-related events were similar between the short- and long-course treatment groups, while short-course treatment was associated with lower mortality (RR = 0.52 [95% CI, 0.33 to 0.82]) and fewer serious adverse events, which mainly included death, life-threatening events, and the prolongation of hospital stay or need for hospitalization (RR = 0.73 [95% CI, 0.55 to 0.97]) compared to long-course treatment; hence, this meta-analysis suggests that short-course antibiotic treatment (≤6 days) is as effective as and potentially superior to, in terms of mortality and serious adverse events, longer-course treatment [39].

An updated meta-analysis published in 2023, assessing the optimal treatment duration of antibiotics for CAP in adults, included nine RCTs (a total of 2399 patients), which evaluated the same antibiotic with the same daily dosage [64]. The results of this duration–effect meta-analysis demonstrated that longer antimicrobial duration resulted in a lower probability of clinical improvement on day 15 and 30, while a shorter treatment duration (3–9 days) was likely to be non-inferior to a 10-day treatment [64]. Also, there was no significant association between harmful outcomes, such as all-cause mortality and severe adverse events, and the duration of antimicrobial treatment [64]. The clinical improvement rates on day 15 in the 10-day treatment arms was 68%, while clinical improvement rates of 3-, 5-, and 7-day treatments were 75%, 72%, and 69%, respectively [64]. A shorter duration (3–5 days) was found to probably achieve the optimal balance between efficacy and treatment burden for treating CAP in adults if they achieved clinical stability [64]. These findings are in line with the previous meta-analyses and current guidelines, which recommend a shorter antimicrobial duration of 5–7 days in patients with CAP [10,28,37,39]. Finally, one systematic review failed to identify any RCTs that compared short and long courses of the same antibiotic for the treatment of adult outpatients with CAP, and thus, this issue remains unclear, and an update of this review should be performed in the future [8].

In summary, the studies comparing short- versus long-course antibiotic therapy in adult CAP patients indicate the non-inferiority of shorter courses compared to longer antibiotic courses, regarding efficacy and safety. However, the majority of the RCTs comparing short- versus long- course antibiotic therapy included patients with mild to moderate or moderately severe CAP (Table 3). Therefore, the optimal duration of antibiotic treatment for CAP is influenced by several factors, with severity of the disease, the type of pathogen, and the presence of complications playing critical roles. While shorter antibiotic courses (typically 5 to 7 days) are often sufficient for mild to moderate cases, severe pneumonia, particularly in patients with co-morbidities or complications, may necessitate prolonged therapy. Hence, in clinical practice, for most cases of uncomplicated, mild to moderate CAP, clinical evidence supports a short course of antibiotics, typically 5 to 7 days, which shows significant clinical improvement. This approach helps reduce the risk of antibiotic resistance and minimizes side effects. However, in patients with severe CAP, those requiring hospitalization or intensive care, or those with complications such as empyema extra-pulmonary complications or bacteremia, a longer course of antibiotics, typically 7 to 14 days, may be warranted to ensure full resolution of the infection. Clinicians should also consider extending treatment in patients with underlying co-morbidities or immunosuppression, where recovery may be slower, or in patients with CAP caused by specific pathogens, such as Staphylococcus aureus, *Pseudomonas aeruginosa*, or atypical bacteria. Regular reassessment of clinical stability is essential to guide the appropriate duration of therapy. In addition, the growing body of evidence in this area has led to a gradual shift in clinical guidelines, which have updated the recommendations to endorse shorter antibiotic courses, especially in patients with mild to moderate CAP. Also, these findings reshaped clinical practices, minimizing antibiotic use, particularly in light of the global issue of antibiotic resistance. However, the small number of existing studies and the overall moderate-to-high risk of bias may compromise the certainty of the results. Thus, although the current evidence strongly favors the “shorter is better” approach, further research on the shorter duration and the specific circumstances under which shorter courses are most appropriate is required.

#### 3.1.2. Shorter Course of Antibiotic Treatment for CAP in Real-Life Clinical Practice

A retrospective cross-sectional study was conducted in a secondary care setting in the United Kingdom (UK), in order to evaluate the optimal antibiotic treatment durations—shorter (≤5 days) versus longer (6–7 days and >8 days)—for respiratory tract infections, in accordance with local antimicrobial guidelines [14]. In this study, a total of 262 patients with CAP were included; 199 of them were treated for shorter durations of ≤5 days, 38 for 6–7 days, and 25 for more than 8 days. A statistically significant difference in the effectiveness and appropriateness of antibiotics was observed across the three duration groups (18.1% for shorter (≤5 days) vs. 22.9% for longer (6–7 days) vs. 20.6% for treatment duration >8 days, *p* = 0.02) [14]. Several studies indicated that the clinical application of the currently available evidence-based guidelines for shorter antibiotic courses in patients with CAP resulted in shorter hospital length of stay, a decline in antibiotic use and duration of all broad-spectrum treatment, MRSA and antipseudomonal therapy, and consequently, in the lower incidence of antimicrobial resistance development [13,65]. Finally, a retrospective multicenter study conducted in Switzerland showed that the duration of antibiotic therapy was longer than recommended by international guidelines in a significant proportion (32%) of CAP patients [66]. The multivariate analysis demonstrated that parameters independently associated with a longer antimicrobial duration than recommended in international guidelines were positive blood cultures, infectious disease consultation, impaired renal function, and an increased C-reactive protein, but not procalcitonin on admission [66].

However, data about the impact of PCT in the duration of antimicrobial treatment in adults with CAP are controversial. Two RCTs demonstrated that PCT-guided initiation and discontinuation of antibiotic therapy had no effect on the duration of antimicrobial treatment, as there was no statistically significant difference between clinical assessment- and procalcitonin-guided groups [67,68]. On the other hand, two other RCTs on the role of PCT in the duration of antimicrobial treatment of CAP showed that the antimicrobial duration was 2–7 days shorter in the PCT-guided antibiotic discontinuation strategy compared to the usual clinical practice [69,70]. A recent randomized controlled trial demonstrated that both CRP- and/or PCT-based algorithms for guiding the discontinuation of antibiotic therapy significantly reduced antibiotic exposure over a 30-day follow-up period, compared to standard care, in patients hospitalized with CAP in non-ICU wards [71]. In addition, it has been reported that PCT-guided antibiotic discontinuation might be useful for shortening the duration of antibiotic treatment without increasing pneumonia recurrence within 30 days after antibiotic discontinuation, especially in patients with severe pneumonia or those with elevated PCT levels on admission [72]. Finally, it has been reported that procalcitonin guidance along with standard guidelines resulted in lower rates of antibiotic prescription, antibiotic exposure, and antibiotic-associated adverse effects [69,70,73,74]. In a real-world study, the use of PCT guidance resulted in shorter overall antibiotic treatment durations and reduced inpatient length of stay, without an impact on hospital readmission rates [75]. In particular, patients with PCT levels < 0.25 µg/L received a shorter mean duration of therapy compared with patients with levels > 0.25 µg/L (4.6 vs. 8.0 days; *p* < 0.001), as well as reduced hospital length of stay (3.2 vs. 3.9 days; *p* = 0.02) [75]. However, data about PCT guidance in real-world settings for the management of pneumonia are limited. The clinical usefulness of PCT-guided antibiotic discontinuation, especially in patients with non-severe pneumonia and those with low PCT levels on admission, needs investigation. Future randomized, controlled, multicenter studies should focus on the non-inferiority of PCT- or CRP-based approaches with respect to clinically relevant patient-centered outcomes.

### 3.2. Hospital-Acquired Pneumonia

HAP is a significant and potentially serious condition that is responsible for a great proportion of nosocomial infections worldwide, accounting for 15 to 23% of all hospital-acquired infections [76,77]. It is often associated with higher morbidity and mortality rates due to the complexities of hospital environments and the prevalence of antibiotic-resistant bacteria, and its management remains a key challenge for clinicians [78,79]. Existing evidence on nvHAP is scarce as surveillance systems, epidemiology, and research traditionally focus on VAP due to its severity and frequency in intensive care settings [80]. This underreporting contributes to a lack of awareness about its true incidence and impact. However, the increasing use of new respiratory support devices, such as high-flow nasal oxygen and noninvasive ventilation, has led to a decrease in VAP incidence, while nonventilated HAP’s relative importance is rising [76,81,82].

The incidence of nvHAP is estimated to range from 0.5 to 1.5 cases per 1000 hospital admissions, depending on the population studied and the hospital setting [82,83,84]. Some studies suggest that nvHAP may occur in about 1% to 2% of all hospitalized patients [78,85]. Notably, a large US study revealed an incidence of 3.63 nvHAP per 1000 patient-days [78]. Moreover, nvHAP has comparable mortality with VAP and a substantial influence on healthcare costs with increased total hospital charges as nvHAP is more prevalent across the entire hospital population and it affects a broader group of patients, including those in general wards [23,76,78].

Common causative agents of HAP can be diverse and include Gram-negative bacteria, such as *Pseudomonas aeruginosa*, Escherichia coli, Klebsiella pneumoniae, Enterococcus coli, Acinetobacter baumannii, and Enterobacter species, and Gram-positive bacteria, such as Staphylococcus aureus and Streptococcus pneumoniae [86]. The vast majority of HAP is caused by multidrug-resistant (MDR) pathogens such as MRSA, MDR strains of *P. aeruginosa*, MDR Acinetobacter species, Extended-Spectrum Beta-Lactamase (ESBL)-Producing Enterobacteriaceae, and carbapenem-resistant Enterobacteriaceae (CRE), posing a significant challenge to clinicians due to the limited treatment options and the potential for severe outcomes [87,88]. Consequently, healthcare providers should use antibiotics judiciously, balancing between the need to treat infections effectively and prevent further antibiotic resistance.

#### Evidence on Antibiotic Treatment Duration of HAP

One of the most vital aspects of managing HAP is determining the optimal duration of antibiotic therapy, which is crucial for improving healthcare outcomes, minimizing side effects, and decreasing antibiotic resistance risk. In the face of rising antimicrobial resistance, recent evidence suggests that short-course antibiotic treatment in patients with common bacterial infections including HAP may be as effective as longer-duration treatment, provided the patient shows clinical improvement [89,90,91]. Healthcare providers often adopt data from VAP management to treat nvHAP patients as there are scant studies comparing short to prolonged antibiotic nvHAP therapy courses. The first study was conducted by Singh et al., who included a case-mix of an ICU source population with suspected VAP and HAP patients (Clinical Pulmonary Infection Score (CPIS) below seven), and they were randomized to receive either standard treatment with broad-spectrum antibiotics for 10–14 days or ciprofloxacin monotherapy with discontinuation after 3 days if pulmonary infiltrate progression was not observed and cultures were negative [61]. The mortality rate did not differ between the two arms, while a shorter length of ICU stay and lower total antimicrobial therapy cost were reported in the restrictive antibiotic treatment group. Additionally, superinfections, infections with MDR pathogens, and antimicrobial resistance were documented in significantly fewer patients who received the short-course strategy (15 vs. 35%, *p* = 0.017) [61].

A retrospective study which included 79 patients with Gram-negative HAP confirmed that short-course treatment (5 days) was not associated with a higher mortality or recurrence rate when pneumonia resolution was achieved prior to therapy cessation [92]. Of these patients, only 21% did not receive mechanical respiratory support, but the findings are not separated between VAP and HAP patients, so the results concern both populations. Among the included patients, at the end of 5 days of therapy, there were only two patients with clear evidence of pneumonia non-resolution, while 14% of cases were documented with recurrence due to relapse or re-infection, of which 80% received mechanical ventilation. The relapse rate was significantly higher among patients with primary HAP attributed to non-fermenting Gram-negative bacilli compared to patients with other Gram-negative organisms (17 vs. 2%) [92]. Consequently, HAP due to NF-GNB is more resistant to eradication, especially when associated with mechanical respiratory support, having a higher risk of recurrence following short-course therapy, and it may require longer antimicrobial treatment. However, this is a limited study and further investigation with RCTs is needed in order to define the appropriate antibiotic therapy duration for nvHAP due to NF-GNB.

The principle of “shorter is better” was investigated in two recent retrospective studies which compared the effectiveness of antibiotic treatment durations, shorter versus longer, for patients with nvHAP [14,93]. Firstly, a study conducted in a UK secondary care setting revealed that shorter antibiotic treatment duration of ≤5 days for HAP was as effective as a longer duration of 6–7 days and >8 days [14]. Moreover, short-course antibiotic therapy was correlated with higher discharge rate and shorter hospital length of stay. In the same context, Tan et al. reported equivalence in the clinical outcome between patients who received brief (5–7 days) and prolonged antibiotic courses (10–14 days) [93]. Clinical resolution was similar between the two groups (69.6 vs. 70.9%), and no difference was observed in mortality rate after 30 (17 vs. 14.5%) and 90 days (20.5 vs. 21.5%) [93]. Nevertheless, a higher superinfection rate was documented in the prolonged-course group compared to the short-course group (6.3 vs. 18.2%, *p* = 0.027), with patients who had nvHAP caused by NF-GNP exhibiting the highest rate of superinfection. Thus, administering the minimum effective duration of antibiotic therapy is essential to reduce the spread of resistant bacteria, while antibiotic de-escalation should be performed as soon as possible based on culture results to minimize the risk of antimicrobial resistance and drug toxicity. Indeed, in a retrospective cohort study that included 279 cases with culture-negative HAP, patients who received <5 days empirical MRSA antibiotic coverage had similar 28-day mortality compared with patients who received >5 days anti-MRSA agent (23 vs. 28%), while anti-MRSA agent de-escalation was associated with shorter hospital length of stay and lower acute kidney injury incidence [94].

The PCT-guided algorithm is one of the most studied strategies for determining antibiotic treatment initiation and discontinuation in pneumonia while minimizing unnecessary antibiotic exposure without altering patient prognosis [95]. While numerous biomarkers for pneumonia have emerged over the past decades, only PCT has been assessed as part of antibiotic de-escalation/discontinuation protocols. Recently, the PROPAGE RCT study demonstrated that measuring PCT levels between day 4 and day 6 in patients with HAP could offer valuable insights into the optimal duration of antibiotic therapy [96]. In detail, 117 elderly patients who had initiated antibiotic therapy for HAP were randomized to receive either an antibiotic regimen that was tailored according to clinical evaluation algorithms guided by PCT levels or a conventional antibiotic regimen that was terminated according to the treating physician’s discretion [96]. The median duration of antibiotic therapy was significantly shorter for the PCT-guided group (8 vs. 10 days, *p* = 0.001) without difference in the recovery rate (84% vs. 89.5%) [96]. Moreover, PCT levels may contribute as biomarkers of clinical efficacy at an earlier stage of the disease. It has been indicated that lower PCT levels on days 3 and 7 and greater rates of procalcitonin decline between days 0 and 3 in patients with HAP were good predictors of treatment response [97]. Although further studies are needed to assess the utility of the daily monitoring of PCT levels and the exact threshold of PCT for predicting therapeutic efficacy in nvHAP patients, optimization of antibiotic treatment duration may be guided by measuring this biomarker early in the disease course, achieving the antimicrobial stewardship goal.

Collectively, although the number of studies which examine the optimal duration of antibiotic treatment for nvHAP is limited, it seems that brief antibiotic regimens are as effective as longer courses with lower rates of superinfection, antimicrobial resistance, and drug toxicity, shorter hospital length of stay, and reduced healthcare costs. Specifically, an antibiotic treatment duration of 5–7 days seems to be appropriate for nvHAP that is in line with international guidelines. Currently, there is not enough evidence to recommend an antibiotic treatment course of less than 5 days. However, the duration should be tailored to the individual patient, taking into account factors such as the causative pathogen and clinical response. The absence of effective methods for monitoring therapeutic efficacy has prompted efforts to focus on biomarkers. The validation of new biomarkers is crucial, and future research should be directed towards the clinical benefits of incorporating biomarker-guided practices into patient care. Randomized controlled trials and high-quality studies are needed to confirm these findings and support the shorter-course approach, bridging the knowledge gaps in the existing literature.

### 3.3. Ventilator-Associated Pneumonia

In the ICU setting, VAP represents an important cause of morbidity and mortality for intubated patients and leads to increased ICU stay, time to extubation, antibiotic consumption, and healthcare costs [25,98]. Diagnostic performance of currently used criteria is characterized by variability and low specificity, while microbiology cultures cannot differentiate between colonization and true VAP [99,100,101,102]. Randomized clinical trials and meta-analyses have investigated whether a shorter course of antibiotics for the treatment of VAP could help to reduce unnecessary antibiotic exposure and antibiotic-associated adverse events, without negatively influencing mortality or infection relapse/recurrence rates [43,45,103]. Other studies have interrogated the effect of using clinical criteria and/or biomarkers to guide early antibiotic discontinuation in patients with VAP [104,105]. While major guidelines now recommend 7–8 days of antibiotic treatment in uncomplicated VAP, the treatment duration of VAP associated with non-fermenting, and often multidrug-resistant, Gram-negative microorganisms (e.g., *Pseudomonas* spp., *Acinetobacter* spp., *Stenotrophomonas maltophilia*) is still debated [22,29,106,107]. Another area of investigation concerns the effectiveness of the application of clinical and/or biomarker (mainly procalcitonin) indices in order to guide early antibiotic cessation in specific subpopulations [108,109]. Moreover, early-onset VAP (occurring within 4–5 days of hospitalization) has been associated with more favorable outcomes compared with late-onset VAP, possibly due to more antibiotic-susceptible causative pathogens and differences in underlying risk factors and may provide an opportunity for shorter treatment duration when clinical improvement is apparent [29,110].

#### 3.3.1. Treatment Duration of VAP Caused by Non-Fermenting Gram-Negative Bacteria

In 2005, ATS/IDSA guidelines recommended a short course (7–8 days) of antibiotics over the previously recommended prolonged course (14–21 days) for the treatment of VAP, with the exclusion of NF-GNB VAP, based on the findings of the PneumoA RCT [44,111]. In this trial, researchers found no difference in mortality and recurrent VAP between the groups treated with the short and the long antibiotic regimen, while antibiotic-free days were higher in the short course [44]. The study also highlighted a higher recurrence rate of VAP caused by NF-GNB in those receiving the short course of antibiotics (mean difference, 15.2%; 90% CI, 3.9% to 26.6%), despite the lack of significant difference in mortality and other secondary endpoints between the two arms [44]. However, the way in which the researchers measured the outcome of VAP recurrence has been under scrutiny, since the short-course group had more days-at-risk for recurrence (21 vs. 14 days). When days at risk were accounted for, the difference in VAP recurrences was not significant [112]. Moreover, recurrence was defined using microbiological-only criteria, and therefore, at least some of the documented episodes could be attributed to colonization instead of true infection [24].

Two subsequent meta-analyses also showed no differences in mortality and other outcomes between short and long courses of antibiotics when considering all cases of VAP [43,45]. For the subgroup of VAP due to NF-GNB, one meta-analysis showed an increased risk of recurrence in the short-course group (OR 2.18; 95% CI 1.14 to 4.16), but not for other outcomes, while showing more antibiotic-free days and less recurrences due to multidrug-resistant pathogens [43]. Based on this evidence and a meta-analysis performed by the panel that showed no difference between the short and long course of antibiotics in pneumonia recurrence for NF-GNB VAP (OR, 1.42; 95% CI, 0.66 to 3.04), the 2016 IDSA/ATS guidelines recommended that patients with VAP should receive a 7-day course of antibiotics, regardless of the presence of NF-GNB. However, the panel also suggested that there are cases that may require shorter or longer regimens depending on the available clinical, radiological, and laboratory data [22]. The 2017 ERS guidelines shared a similar approach in regard to treatment duration for VAP, recommending a 7–8-day course of antibiotics (excluding cases complicated by immunodeficiency, cystic fibrosis, empyema, lung abscess, cavitation, or necrotizing pneumonia) for patients showing a good clinical response to treatment [29]. Subsequently, the first RCT that would put to test the short versus long course of antibiotics in exclusively *Pseudomonas aeruginosa* (PsA)-VAP was prematurely terminated due to inadequate enrolment and was unable to prove non-inferiority of the short course for the composite outcome of 60-day mortality and PsA-VAP recurrence [59]. Despite being underpowered, the study did not find differences between the 7- and 15-day course in the composite and the secondary outcomes, except for a trend for higher VAP recurrence in the short-course group (17% vs. 9.2%, MD 7.9%; 90% CI −0.5 to 16.8%) [59]. Two recent meta-analyses, which both included this RCT, showed no difference in 28-day mortality or ICU length-of-stay between the short and the long course of antibiotics, while finding more antibiotic-free days in the short course [103,113]. Regarding VAP recurrence/relapse (Figure 4), one of these two meta-analyses found a higher rate for the short treatment group, which was primarily driven by NF-GNB VAP [113]. A recent study by Mo et al., including a patient population with VAP and high representation of NF-GNB and MDR organisms, found non-inferiority of an individualized, clinically guided short course compared to usual care [60]. For the subgroup of NF-GNB, the investigators did not find significant differences between the two arms regarding the primary outcome (OR 1.38; 95% CI 0.65 to 2.92) [60]. When all evidence is considered, one has to balance between the possibility of higher recurrence rates with shorter courses of antibiotics and the documented benefits of this strategy, i.e., more antibiotic-free days and less antibiotic-related side effects.

#### 3.3.2. Procalcitonin to Guide Shorter Course of Antibiotic Treatment for VAP

Procalcitonin has been investigated in several observational studies and RCTs as a biomarker to predict the response to antibiotic treatment and guide early antibiotic cessation in VAP [41,108]. A meta-analysis showed that using PCT algorithms to stop antibiotics reduced the duration of treatment from 13.1 to 10.8 days (mean difference −2.22 days; 95% CI −3.8 to −0.65, *p* = 0.006), without influencing mortality, treatment failure, and ICU length of stay (LOS) [114]. The 2016 IDSA/ATS panel group pooled the results of three RCTs and found similar results for antibiotic discontinuation based on PCT plus clinical criteria versus clinical criteria alone [22]. When the recommended 7 to 8 days of treatment is followed, the added benefit of using PCT to further reduce antibiotic exposure is debatable. Similarly, the ERS guidelines recommend against using PCT for this purpose, while considering its use when a longer treatment duration is expected, e.g., presence of immunosuppression, MDR pathogens, and optimal treatment not available [29]. Another study investigated the role of the Clinical Pulmonary Infection Score (CPIS) in conjunction with serial spot PCT measurements after day 7 of treatment, using a cut-off of 0.5 ng/mL to guide antibiotic cessation in the intervention group. The trial, which enrolled only patients that showed adequate clinical response on day 7, showed that CPIS plus PCT increased antibiotic-free days (14.6 vs. 5.9 days, *p* < 0.001), decreased antibiotic treatment for VAP from 13.3 to 8.7 days, and had no impact on VAP recurrence and days on mechanical ventilation [108]. More recently, Mazlan et al. conducted an RCT to assess the value of a point-of-care PCT test to determine antibiotic treatment duration in patients with VAP and found less antibiotic exposure in the PCT arm (10.28 vs. 11.52 days; mean difference −1.25; 95% CI −2.48 to 0.01; *p* = 0.049), with no effect on mortality and retreatment rates [115]. Consequently, PCT may have a role in safely reducing antibiotic exposure and antibiotic-associated side effects in patients with VAP that are expected to need a prolonged course of antibiotics. More studies are needed in order to determine the value of PCT in these specific populations.

#### 3.3.3. Clinical Application of Current Evidence

Current evidence suggests that a 7-day antibiotic course is sufficient for uncomplicated VAP, including NF-GNB VAP [24,116,117]. Nevertheless, inadequate clinical response to treatment, such as continuing fever, presence of purulent sputum, and clinical deterioration, may warrant a more extended antibiotic course, and management in these patients should be individualized [107]. Moreover, most trials evaluating short versus long courses of antibiotics in VAP have not included patients with immunocompromising conditions (human immunodeficiency virus infection, neutropenia, immunosuppressive treatment, and cystic fibrosis) or complicated VAP (empyema, necrotizing pneumonia, and lung abscess). Further, considering the increasing prevalence of MDR/extensively drug-resistant (XDR) pathogens, especially in the ICU setting, fully active antibiotic regimens may not be available, and initial empiric treatment may be inappropriate, while treatment duration for these infections has not been established. Consequently, a short antibiotic regimen may not be feasible for these patient populations. Evaluation of a patient’s response to treatment is primarily clinical but may be accompanied and complemented by clinical scores and/or PCT measurement, especially when a long course of antibiotic treatment is considered.

## 4. Methods

We conducted free searches on the PubMed database from its inception to 15 August 2024, using several combinations of 6 concepts comprising the following keywords (including their related MeSH terms) in order to retrieve as many articles as possible: concept 1: antimicrobials (“antimicrobials” OR “antibiotics”); concept 2: shorter duration (“shorter duration” OR “short-course” OR “shorter course”); concept 3: pneumonia (“pneumonia” OR lower respiratory tract infection” OR “respiratory infection”); concept 4: community-acquired pneumonia (“community-acquired pneumonia” OR “CAP” OR “community-acquired lower respiratory tract infection”); concept 5: hospital-acquired pneumonia (“hospital-acquired pneumonia” OR “HAP” OR “health-care associated pneumonia” OR “HCAP” OR nosocomial pneumonia); concept 6: ventilator-associated pneumonia (“ventilator-associated pneumonia” OR “VAP” OR “ventilator-associated lower respiratory tract infection”). Additionally, we searched references from retrieved articles and guidelines to identify potential articles not captured in our PubMed search. The search was conducted by two reviewers. Papers published in non-English language were excluded as well as conference abstracts and book chapters.

## 5. Conclusions and Future Perspectives

The review of existing literature, and especially the accumulated evidence from RCTs and meta-analyses, suggests that shorter courses of antibiotics may be an effective treatment option for adult patients with pneumonia. Evidence demonstrates that shorter antibiotic regimens can achieve clinical outcomes comparable to the standard of care of longer courses, including similar rates of clinical cure, microbiological eradication, and recurrence and leading to lower rates of antibiotic exposure and resistance, fewer adverse effects, improved patient compliance, decreased hospitalization duration, and lower healthcare costs. As the list of drug-resistant bacteria and fungal infections is steadily increasing and antimicrobial resistance has been globally recognized as an immediate threat, the administration of shorter courses of antibiotics is crucial. The highlighting of the effectiveness of shorter antibiotic courses in patients with pneumonia and their widespread use in clinical practice could contribute to the prevention of this critical issue. At the same time, the development of antimicrobial stewardship programs towards this direction and the continuous reinforcement of the efficacy and benefits of the shorter courses of antibiotics are essential for their application in clinical practice. Eventually, once the recommendations for shorter courses have been disseminated and implemented and the change in practice of the duration of treatment of pneumonia has occurred, full evaluation and an ongoing audit of the changes in practice are required to sustain the changes.

The findings strongly advocate for the integration of shorter antibiotic regimens into international clinical guidelines. However, the adoption of shorter antibiotic courses should be approached with caution. The optimal duration of antibiotic therapy may vary based on several factors, including the severity of the pneumonia, the presence of comorbid conditions, and the specific pathogens involved. Personalized treatment plans, guided by clinical judgment and supported by diagnostic tools and biomarkers, are essential to ensure the efficacy and safety of shorter antibiotic regimens.

Despite the vigorous evidence supporting shorter antibiotic courses as a promising approach to the treatment of pneumonia in adults, further research is needed to optimize their use. Hence, large-scale RCTs should further validate the efficacy and safety of shorter antibiotic courses for different types and severities of pneumonia and refine treatment recommendations, particularly in specific patient populations, such as immunocompromised patients, the elderly, and those with severe pneumonia. Additionally, the development and validation of reliable biomarkers or clinical predictors that identify patients who would benefit from shorter therapy versus those who might require a longer duration of treatment is crucial. Continuous evaluation of these treatment strategies, along with ongoing surveillance of antimicrobial resistance patterns, is important in order to improve patient outcomes and contribute to the global effort to combat antibiotic resistance.

## Figures and Tables

**Table 1:** Definitions of the different types of pneumonia.

Types of Pneumonia	Definitions
Community-acquired pneumonia (CAP)	Pneumonia acquired outside of the hospital or within 48 h after admission [21].
Hospital-acquired pneumonia (HAP)	Pneumonia in patients who have been admitted to the hospital for at least 48 h and did not have relevant symptoms at the time of admission. HAP is further classified as non-ventilator hospital-acquired pneumonia (nvHAP) and ventilator hospital-acquired pneumonia (VAP) [22,23].
Ventilator-associated pneumonia (VAP)	A type of pneumonia developing in patients who are on mechanical ventilation for at least 48 h [22,24,25].

**Table 2:** International guidelines on the optimal duration of antibiotic therapy (in days) for different types of pneumonia.

Guidelines	Year		Duration of Antibiotic Therapy		
	CAP	HAP/VAP	CAP	HAP	VAP
British Thoracic Society (BTS) [26]	2009	N/A	7 days for low/moderate severity without complications (formal combination of expert views)	N/A	N/A
			7–10 days for high severity (formal combination of expert views)		
			14–21 days for *S. aureus* or Gram-negative bacilli (formal combination of expert views)		
National Institute for Health and Care Excellence (NICE) [27]	2019	2019	5 days for all types of severity if clinical stability is achieved	5 days for mild symptoms and lower risk of resistance, then review	N/A
American Thoracic Society (ATS)/Infectious Diseases Society of America (IDSA) [10,22]	2019	2016	Minimum of 5 days for low, moderate, and high severity without complications (strong recommendation based on a small number of RCTs) 7 days for *MRSA* or *P. aeruginosa* (strong recommendation) Longer courses for meningitis, endocarditis or for less-common pathogens (strong recommendation)	7-day antibiotic treatment (strong recommendation based on systematic reviews of RCTs)	7-day antibiotic treatment (strong recommendation based on systematic reviews of RCTs)
European Respiratory Society (ERS)/European Society of Intensive Care Medicine (ESICM)/European Society of Clinical Microbiology and Infectious Diseases (ESCMID)/Asociación Latinoamericana del Tórax (ALAT) [28,29]	2023	2017	Minimum of 5–7 days for severe CAP (sCAP) when clinical stability is achieved with the use of PCT (conditional recommendation based on RCTs) Minimum of 7 days for *S. aureus* CAP (strong recommendation)	7–8 days (good practice statement based on systematic reviews of RCTs)	7–8 days (weak recommendation—moderate quality of evidence) 7–8 days for non-fermenting Gram-negative bacteria, *Acinetobacter* spp., and MRSA with good clinical response
Chinese Thoracic Society (CTS)/Chinese Medical Association (CMA) [30,31]	2016	2018	5–7 days for mild/moderate severity	7 days or longer for immunocompetent patients with good clinical response	7 days or longer for immunocompetent patients with good clinical response
			Prolonged for severe CAP or with extra-pulmonary complications		
			10–14 days for patients with atypical pathogens	Prolonged accordingly for XDR or PDR pathogens	Prolonged accordingly for XDR or PDR pathogens
			14–21 days in the case of *S. aureus, P. aeruginosa, Klebsiella* and anaerobic bacteria		
South African Thoracic Society (SATS) [32,33]	2017	2006	5–7 days for low/moderate severity (strong recommendation based on well-designed studies)	5–7 days	5–7 days
			14 days for *Staphylococcus aureus* bacteremia (strong recommendation based on well-designed studies)		
			7 days for *Legionella* (strong recommendation based on well-designed studies)		
South Australian Department of Health (SA Health) [34,35]	2021		5 days for low severity	5 days minimum and review	5 days minimum and review
			5–7 days for moderate severity		
			7 days for high severity		
			Consult for *S. Aureus* or *Pseudomonas aeruginosa*		

CAP: community-acquired pneumonia; HAP: hospital-acquired pneumonia; VAP: ventilator-associated pneumonia; N/A: not applicable; XDR: extensively drug resistant; MDR: multidrug resistant; MRSA: Methicillin-resistant Staphylococcus aureus; S. Aureus: Staphylococcus aureus; *P. aeruginosa*: *Pseudomonas aeruginosa*; PCT: procalcitonin; PDR: pan-drug resistant; RCTs: randomized controlled trials.

## Data Availability

The data underlying this article will be shared upon reasonable request to the corresponding author.
